# Phase retrieval from single interferogram without carrier using Lissajous ellipse fitting technology

**DOI:** 10.1038/s41598-023-36584-5

**Published:** 2023-06-19

**Authors:** Fengwei Liu, Yu Kuang, Yongqian Wu, Xiaojun Chen, Rongzhu Zhang

**Affiliations:** 1 National Key Laboratory of Optical Field Manipulation Science and Technology, Chinese Academy of Sciences, Chengdu, 610209 Sichuan China; 2 The Institute of Optics and Electronics, Chinese Academy of Sciences, Chengdu, 610209 Sichuan China; 3 Youth Innovation Promotion Association CAS, Beijing, China; 4 School of Electronic and Information Engineering, Sichuan University, Chengdu, 610065 China

**Keywords:** Optical techniques, Optical physics

## Abstract

Phase extraction from single interferogram is of high significance and increasingly interest in optical metrology. In this contribute we propose an advanced Pixel-level Lissajous Ellipse Fitting (APLEF) method to extract the phase from single interferogram without carrier. At each pixel, a Lissajous figure is created by plotting *N* against *D*, where *N* and *D* are subtractions and additions of intensities of adjacent pixels in a small window. The so created Lissajous figure is already in phase quadrature because of the subtraction and addition process, and the Lissajous Figure is forced to be closed by taking the opposite values of *N* and *D,* i.e. *–N* and *-D* into account. The closed and in phase quadrature Lissajous Figure is the key point for APLEF to demodulate the single inteferogram without carrier in theoretically. The simulation shows its higher accuracy than existed SPT and Garbusi’s method and the experiments finally corroborate its effectiveness.

## Introduction

It is a general problem to demodulate 3-D phase information from the recording 2-D intensity distributions in optical metrology. Temporal phase-shifting (TPS) interferometry is the most accurate one^1,2^ and is typically used to measure static physical quantity, e.g., optical surface error. However, on various occasions such as environmental chambers, airflow analysis, and Meter-class optics testing, the environment is dynamically changing^3^, TPS is no more appropriate. It is urgent to develop dynamic phase measurement technique. Single interferogram demodulation technique is a straightforward candidate for one-shot exposure can freeze the vibration and air disturbances. Both Fourier Transform method (FTM)^4–7^ and spatial carrier-frequency phase shifting (SCPS)^8–10^ are well established techniques for this purpose, whereas the spatial phase carrier is necessary and is supposed to be enough to force the monotonic behavior of the encoded phase.

On occasions that carrier cannot introduce, e.g., at the early stage of optics polishing carrier may directly result in unresolved fringes, we may have to face the problem of estimating phase from single interferogram with possibly closed fringes. However, it is challenging in practical. The trickiest problem lies in the undetermined phase sign for the intrinsic even character of cosine function, that is cos (−φ) has the same results with cos (φ) so one can’t determine the exact sign of φ from the intensity without a priori.

The regularized phase-tracking (RPT)^11^ proposed by M. Servin in 1997, and the frequency-guided sequential demodulation (FSD) method^12^ proposed in 2006 are all able to demodulate the single interferogram without spurious phase sign by incorporating with the smoothness constraints of the phase. However, the sacrifice is extremely large time expenditure for minimization of a regularized cost function. Besides, they all need complicated fringe normalization process in prior.

In 2001, Larkin proposed spiral phase transform (SPT) method^13,14^. It is actually a successful application of 2-D Hilbert transform for the fringe pattern. The 2-D quadrature signal of the closed-fringe interferogram without sign ambiguous is obtained using SPT aided by a fringe direction map. However, the fringe direction estimation is not a trivial process, which needs very carefully calculation^15–19^.

Another method deserves to be mentioned is proposed by E. Garbusi in 2008^20^, A temporal phase shifting algorithm (PSA), i.e., Carre algorithm is applied in a spatial fashion for single closed-fringe interferogram demodulation. Carre’s algorithm is much more sensitive to errors especially the phase shift/carrier is small, and there has no detail description for the ambiguous phase sign correction.

In this paper, we propose an advanced Pixel-level Lissajous Ellipse Fitting (APLEF) method to demodulate the single closed-fringe interferogram. It is also based on a spatial scanning strategy. One advantage of proposed APLEF is no need for complex fringe normalization, which is preferable over most phase tracker methods. In addition to that, proposed APLEF has no careful scanning map required and the computation loads are relatively less. Last but not least, we use image segmentation to guide the spurious phase sign correction which is a straightforward way and more tolerant to noise than fringe orientation map estimation.

In the next section, the principle of APLEF will be explained, and the intensity rearranging strategy is then proposed. The image segmentation for spurious phase sign correction is also illustrated briefly.

## Methods

The intensity of a fringe pattern can be written in Eq. (1)

1 $$ I(x,y) = A(x,y) + B(x,y) \cdot \cos [\varphi (x,y)] + n(x,y) $$

where $A(x,y),B(x,y)$ respectively represent the background intensity and the modulation amplitude of the fringe. $\varphi (x,y)$ is the target phase and $n(x,y)$ is the additive noise.$(x,y)$ means the spatial location of each pixel and will be saved for simplicity.

Before the phase demodulation, the low frequency background, and high frequency noise $n(x,y)$ are suggest to be filtered. 2-D Hilbert-Huang transform method^21^ or Variational Image Decomposition (VID)^22,23^ are good candidate for this purpose.

In a small working window of the fringe pattern, we assume the local phase $\varphi$ is linear and the modulation $B$ is a constant. With the assumptions above, the intensities of any two adjacent pixels in the working window can be expressed as:

2 $$ \overline{I}_{1} = B \cdot \cos \left( \varphi \right),\overline{I}_{2} = B \cdot \cos \left( {\varphi + \delta } \right) $$

where $\delta$ means the phase difference or gradient between the two adjacent pixels, it is supposed to be a small and constant value (typically less than 0.1 rad).

With addition and subtraction of intensities of two adjacent pixels we can derive Eq. (3):

3 $$ I_{a} = \overline{I}_{1} - \overline{I}_{2} { = }2B \cdot \sin \left( {\delta /2} \right)\sin \left( {\varphi + \delta /2} \right), I_{b} = \overline{I}_{1} + \overline{I}_{2} { = }2B \cdot \cos \left( {\delta /2} \right)\cos \left( {\varphi + \delta /2} \right) $$

The following Eq. (4) is the general expression of Eq. (3) and is also the parametric form of an ellipse^24^.

4 $$ N\left( \phi \right) = a_{x} \sin \left( \phi \right), D\left( \phi \right) = a_{y} \cos \left( \phi \right) $$

Here $$\phi = \varphi + \delta /2$$,$$a_{x} = 2B \cdot \sin \left( {\delta /2} \right)$$ , $$a_{y} = 2B \cdot \cos \left( {\delta /2} \right)$$.

Finally, we can calculate the phase of the working pixel by Eq. (5) once the parameters of Eq. (4) are achieved.

5 $$ \varphi = \phi - \delta /2\begin{array}{*{20}c} {} & { = \tan^{ - 1} \left( {\frac{{a_{y} }}{{a_{x} }} \cdot \frac{N}{D}} \right) - \tan^{ - 1} \frac{{a_{x} }}{{a_{y} }}} \\ \end{array} $$

**Figure 1:**
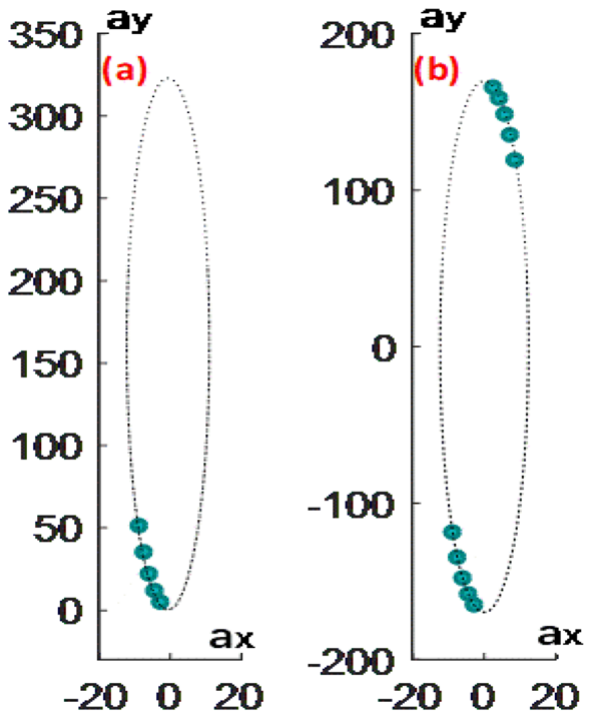
Lissajous figure and fitted ellipse without (**a**) and with (**b**) intensity rearranging.

We use Lissajous Figure and Ellipse Fitting technique^24–27^ to calculate the parameters of Eq. (4), i.e., $a_{x} ,a_{y}$. Equation (4) clearly shows the created Lissajous figure is already in phase quadrature, which can ease the ellipse fitting process. However, it should be highlighted the Lissajous figure is open since phases of the selected pixels vary much gentle in the working window of a non-carrier interferogram. Figure 1a shows an open Lissajous Figure possibly like. To fit an open Lissajous Figure with noise accurately, the ellipse fitting algorithm (EFA) needs to be robust enough. Hyper ellipse fitting algorithm (HEFA)^28^ is a good choice, whereas it is still inadequate since only a few points are available to create the Lissajous figure. Therefore, we must explore another strategy to strengthen the robustness of EFA.

Taking the opposite values of $I_{a} ,I_{b}$ we will have the following equations

6 $$ - I_{a} = 2B \cdot \sin \left( {\delta /2} \right)\sin \left( {\varphi + \delta /2 + \pi } \right), - I_{b} = 2B \cdot \cos \left( {\delta /2} \right)\cos \left( {\varphi + \delta /2 + \pi } \right) $$

Obviously, $I_{a}$ and $- I_{a}$ both obey the formula of $N\left( \phi \right) = a_{x} \sin \left( \phi \right)$. $I_{b}$ and $- I_{b}$ obey the formula of $D\left( \phi \right) = a_{y} \cos \left( \phi \right)$ . It means the number of points will be doubled on the created Lissajous figure and more importantly their phase differences are $\pi$, as Fig. 1b shows.

Therefore, the intensity rearranging ensures the created Lissajous figure close. Thus, EFA will be more tolerant to noise.

The phase extracted using Eq. (5) unsurprisingly has sign changes if the interferogram contains closed-fringe. Local phase with different sign in the extracted phase map belongs to different zones which can be detected by the phase discontinuity. Therefore, the spurious phase sign can be corrected if the zones are separated very well. It is naturally to use the edge detection operator such as *Sobel, Prewitte* or *Log* for boundary of phase discontinuity detection. However, it is difficult to accurately detect the boundary at the saddle points or other null phase gradient points (also known as critical points^11^) the detected edges are also discontinued. Here, we propose to use image segmentation to identify the phase change areas. The segmentation for this model is much easier for deep learning net^29^, cause only two categories are needed. We propose a novel image segmentation model that leverages visual transformers while also incorporating the strengths of convolutional neural networks (CNNs). We conduct a thorough comparative analysis of existing image segmentation models to demonstrate the effectiveness of our approach. Since the theme of this paper is phase demodulation algorithm, more details about the phase sign change map estimation based on image segmentation will be described in a following paper.

## Discussion

### Simulations

**Figure 2:**
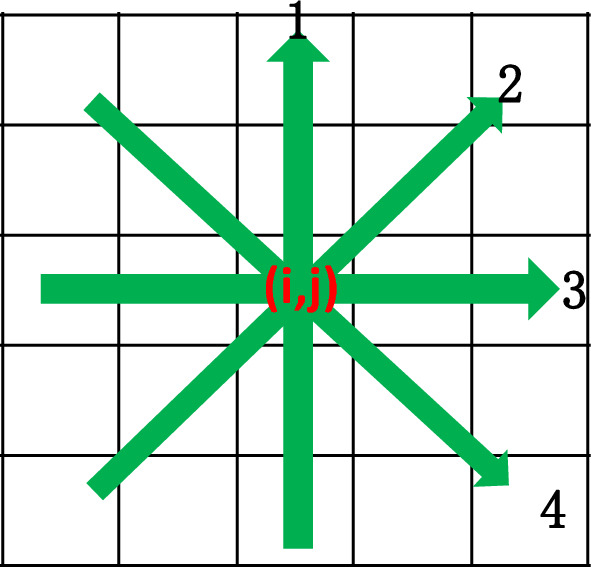
The schematic of fringe orientation.

A series of interferograms with different assumptions are produced according to the fringe model, i.e., Eq. (1) to test the ability of proposed method, meanwhile SPT and Garbusi’s spatial method are also analyzed for a straightforward comparison. It is important to clarify three points in advance. First, the iterative correction process around the critical points of Garbusi’s method is not included in this comparison; Second, the fringe direction map estimation in SPT is performed by the principal component analysis (PCA) based method proposed by Zhang and Guo in^16,17^; Third, the phase sign change map for APLEF and Garbusi’s method is estimated by directly performing the phase difference calculation along the fringe orientation. Here, the fringe orientation at pixel (i, j) is simply defined as the maximum intensity difference along 1,2,3,4 in Fig. 2

**Figure 3:**
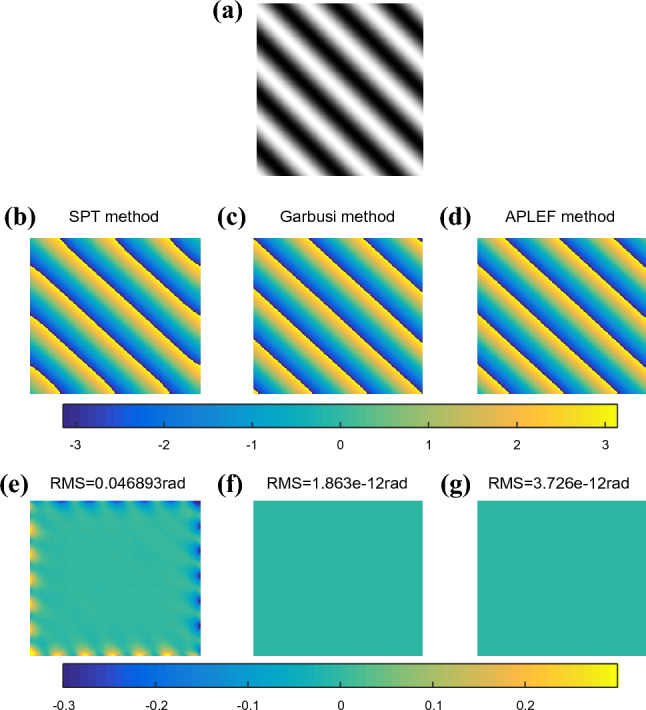
The phase demodulation of a single interferogram with small carrier (**a**) using SPT (**b**),Garbusi’s method (**c**), APLEF method (**d**), and the corresponding phase extraction error maps (**e**–**g**).

In the first situation, we assume the modulation is constant in a small window and the local phase is linear. It is actually the ideal model, where only the cosine term of the fringe model is considered and the encoded phase is a pure tilt. Here the tilt results in a carrier frequency of only 0.2rad/pixel in 45 degree as Fig. 3a shows. After the demodulation process of SPT, Garbusi’s method and proposed APLEF method, we achieved the respective wrapped phases in Fig. 3b–d and corresponding extraction error maps in Fig. 3e–g. It clearly shows that as a frequency domain method, SPT has obvious Gibbs effect at the edge (discontinuity) while the spatial domain methods, namely APLEF and Garbusi’s method are almost error free. It demonstrates the greater potential of spatial phase demodulation method than frequency domain method.

**Figure 4:**
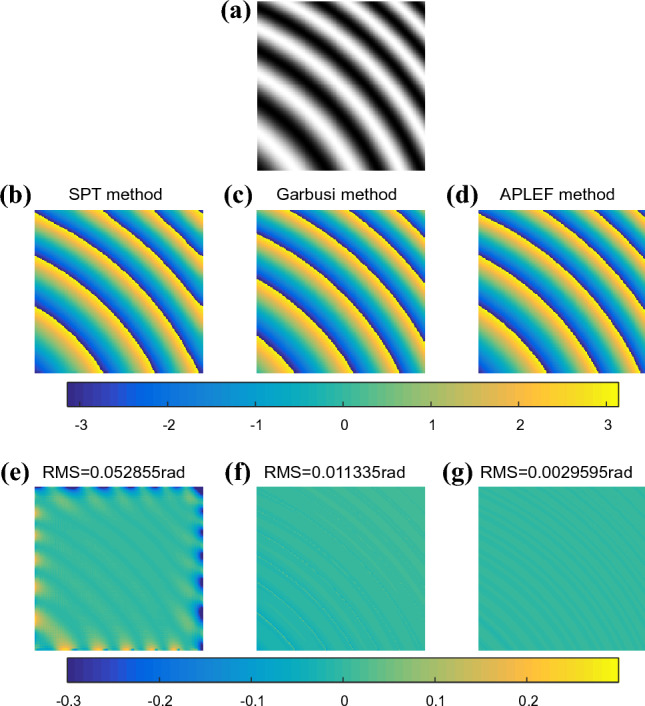
Demodulating a defocus from a single interferogram with small carrier (**a**), using SPT (**b**), Garbusi’s method (**c**), APLEF method (**d**), and the corresponding phase extraction error maps (**e**–**g**).

In the second situation, we assume the encoded phase is a defocus error (PV = 2 rad) with a carrier of 0.2 rad/pixel in 45 degree. The fringe is shown in Fig. 4a. Figure 4b–g display the demodulated wrapped phases and the extraction error maps of SPT, Garbusi’s method and APLEF method. The nonlinear local phase means unequal local phase shift thus resulting in detuning error of the spatial domain methods. APLEF, the extraction error of which is 0.003 rad, has less sensitivity to the detuning error comparing to Garbusi’s method, the extraction error of which is 0.011 rad as Fig. 4f–g shows. For SPT, the Gibbs effect error, shows in Fig. 4e is still the dominate error source.

**Figure 5:**
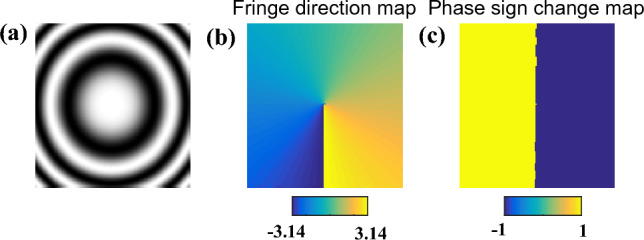
A single interferogram with closed fringe (**a**), the estimated fringe direction map by PCA based method (**b**), the phase sign change map calculated with the true phase (**c**).

**Figure 6:**
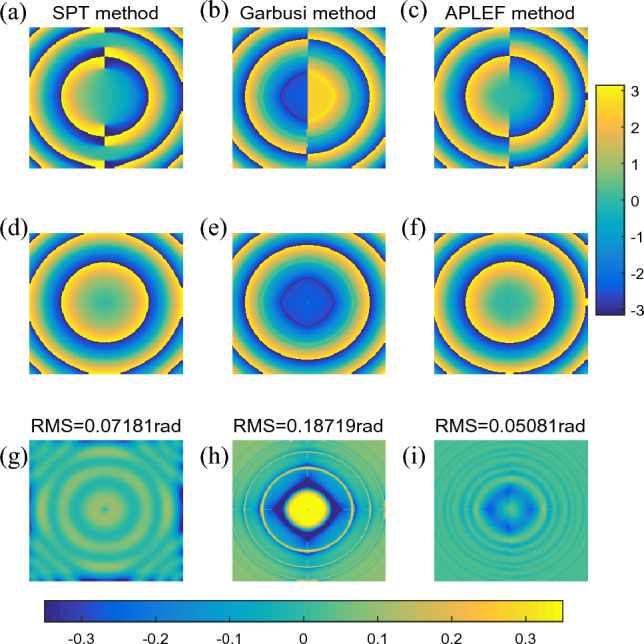
The demodulated wrapped phases with sign ambiguous of SPT (**a**), Garbusi’s method (**b**), APLEF method (**c**), the corresponding wrapped phases with sign corrected (**d**–**f**), and the corresponding phase extraction error maps (**g**–**i**).

In the third situation, the encoded phase is a defocus error (PV =20rad) without any carrier, which means the fringe will be closed as Fig. 5a shows. Before the phase demodulation, we estimate the fringe direction using PCA based method proposed in^16^, which is shown in Fig. 5b. Besides, the phase sign change map in Fig. 5c is also calculated. SPT method, Garbusi’s method and proposed APLEF are respectively applied to demodulate the closed fringe. Figure 6a–c show the extracted wrapped phases of the three methods. It is clear that the demodulated wrapped phases of both frequency domain method and spatial domain methods contain spurious phase sign problem. It is worth to note that the calculated wrapped phase of SPT is erroneous, characteristic phase mismatch error line is observable. That is due to the lack of fringe direction information^30^. However, the spatial domain methods, both Garbusi’s method and APLEF are only phase sign changed. It is the significant difference between proposed APLEF and SPT, and it is also an advantage of proposed method, since the phase sign change map is comparatively easy to achieve comparing to fringe direction map. Thus, for proposed APLEF, the spurious phase sign can be directly corrected by multiplying a binary map as Fig. 5c shows. Figure 6d–f is the demodulated wrapped phase with spurious phase sign corrected. The extraction error maps of the three methods are correspondingly shown in Fig. 6g–i which denote that APLEF has the smallest phase extraction error among the three methods in this situation. We notice that Garbusi’s method exhibits huge error around the null phase gradient point, the reason lies in Carre algorithm is much more sensitive to detuning error especially when the nominal phase shift is too small, e.g. less than 0.1rad as has been demonstrated in^31^, it is also the reason why Garbusi et.al proposed to use an iterative process in^20^ to enhance the accuracy of phase extraction.

**Figure 7:**
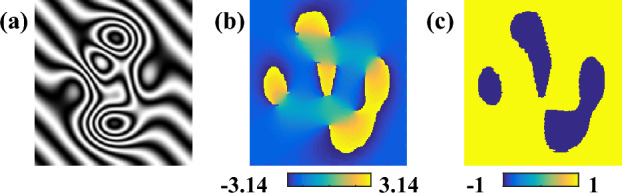
A single interferogram with complex fringe (**a**), the estimated fringe direction map by PCA based method (**b**), the phase sign change map calculated with the true phase (**c**).

In the fourth situation, more complex fringe pattern, e.g., Fig. 7a is created. The encoded phase is a *peaks* function with carrier of 0.3 rad/pixel in 45 degree. We estimate its’ fringe direction map, i.e., Fig. 7b and phase sign change map, namely Fig. 7c in advance to guide the spurious phase sign correction.

**Figure 8:**
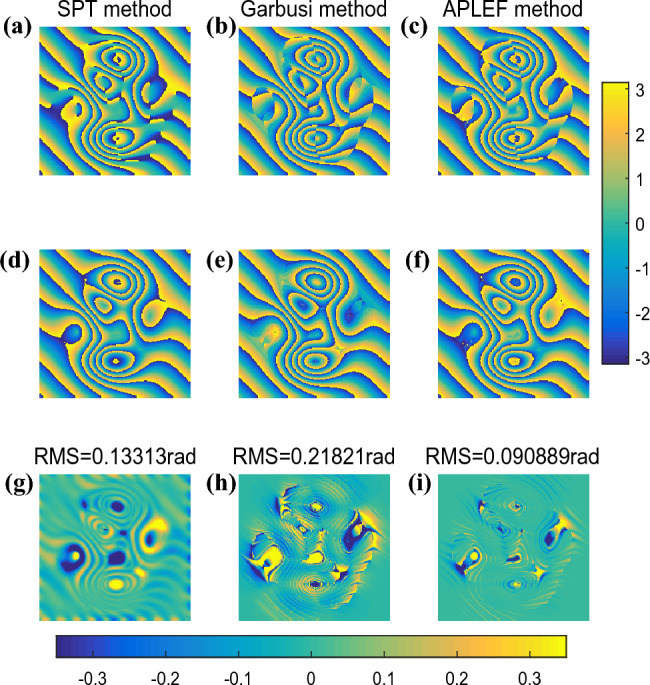
The demodulated wrapped phases with sign ambiguous of SPT (**a**), Garbusi’s method (**b**), APLEF method (**c**), the corresponding wrapped phases with sign corrected (**d**–**f**), and the corresponding phase extraction error maps (**g**–**i**).

The three methods are applied to demodulate the complex fringe pattern once again. Figure 8a–c are the extracted wrapped phases with sign ambiguity of SPT, Garbusi’s method and APLEF.

With the fringe direction map and phase sign change map, we can easily correct the spurious phase sign as shown in Fig. 8d–f. The three methods are all able to extract the phase from single complex fringe pattern, but large phase extraction error emerges around the critical points. In this fact, APLEF has the smallest phase extraction error among the three methods which is less than 0.1rad in RMS, the corresponding extraction error maps of SPT, Garbusi’s method and APLEF are shown in Fig. 8g–i.

**Figure 9:**
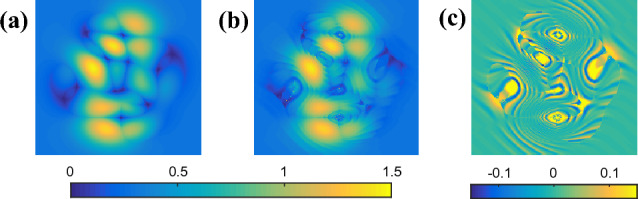
The instantaneous frequency of the complex fringe pattern (**a**), the calculated one by APLEF (**b**), the residual error map (**c**).

Another bonus of proposed APLEF is that, the instantaneous frequency (with sign ambiguous) of the fringe can be simultaneously estimated, which is shown in Fig. 9b. It is useful in other fringe analysis methods, e.g., FSD^12^. By comparing with the nominal instantaneous frequency map, shown in Fig. 9a, we can achieve the residual error map in Fig. 9c, which clearly denotes that, the lower frequency of the fringe it is, the higher extraction error it will be.

**Figure 10:**
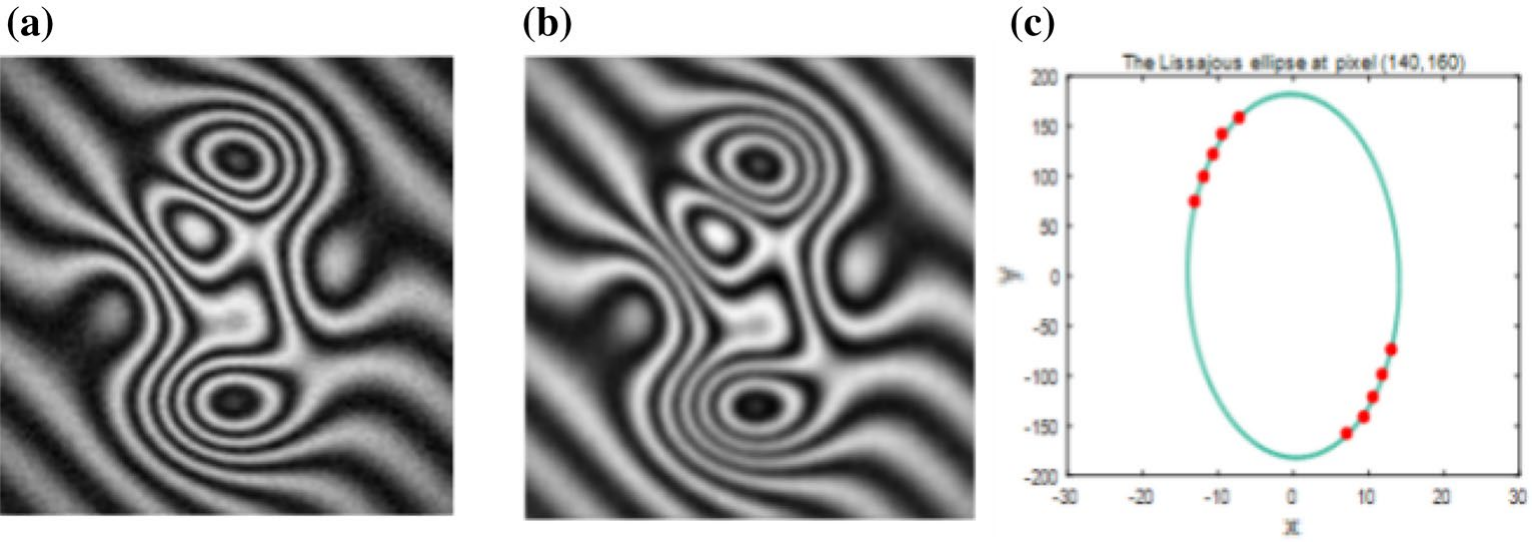
The single complex fringe pattern with non-uniform background and modulation under noisy circumstance (**a**), the pre-filtered fringe pattern with background removed and noise filtered (**b**), the Lissajous ellipse at pixel (140,160) (**c**).

In the above four situations, we only used the cosine term of the fringe model to verify the correctness of APLEF. In reality however, the background, modulation term and also additive noise exist. Therefore, in the fifth situation, we will consider the complete fringe model. Here the background $A$ and modulation $B$ are both obey Gaussian distribution, i.e.

7 $$ G(x,y) = 100\exp \left[ { - k_{g} \cdot (x^{2} { + }y^{2} )} \right];( - 1 \le x,y \le 1) $$

where $k_{g}$ determines the degree of ununiformity and *k*_*g*_ is set to 0.4. 10% additive noise is added by *rand* function. The so created fringe pattern is shown in Fig. 10a. As we introduced in Sect. 2, APLEF doesn’t need fringe normalization but the background (DC term) should be removed. As a pixel-wise demodulating method, APLEF is also sensitive to noise, therefore fringe preprocessing is highly required. Here, a Gaussian high pass filter with sigma = 1 is used for background removal and a Butterworth low-pass filter with radius = 20 is used for noise filtering. The pre-filtered interferogram is shown in Fig. 10b. The Lissajous ellipse at pixel (140,160) of Fig. 10b is shown in Fig. 10c.

**Figure 11:**
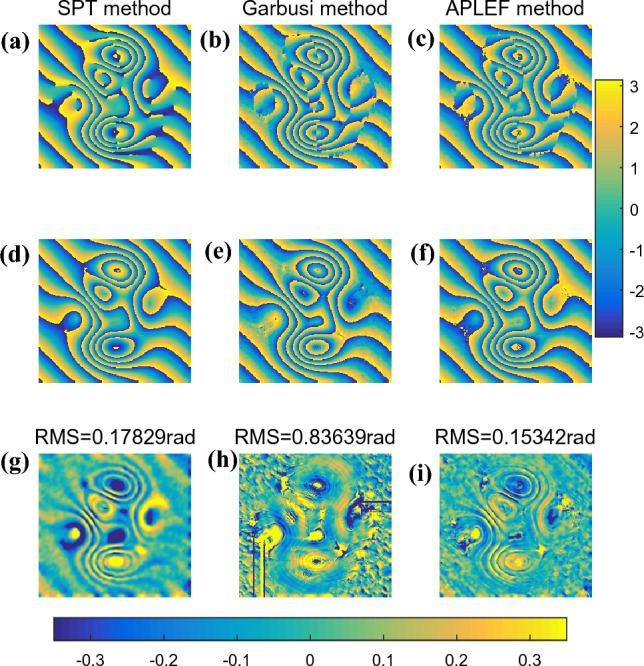
The demodulated wrapped phases with sign ambiguous of SPT (**a**), Garbusi’s method (**b**), APLEF method (**c**), the corresponding wrapped phases with sign corrected (**d**)-(**f**), and the corresponding phase extraction error maps (**g**–**i**).

After the fringe preprocessing, three methods are respectively applied to demodulate the single complex fringe pattern. The demodulated wrapped phases with/without phase sign ambiguous are displayed respectively in Fig. 11a–f. The phase extraction error maps in Fig. 11g–i indicate that even though the noise is pre-filtered, it is still difficult to achieve smooth phase directly for spatial methods like Garbusi’s method. That may cause the phase unwrapping process to fail, as Fig. 11h shows.

**Figure 12:**
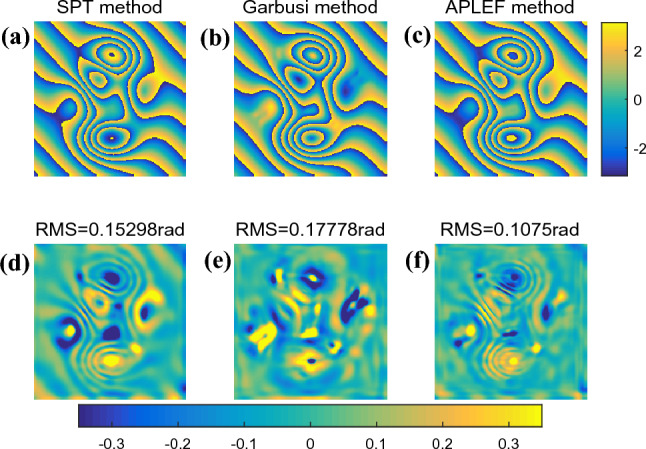
The refined wrapped phases of SPT (**a**), Garbusi’s method (**b**), APLEF method (**c**), using WFF, and the corresponding phase extraction error maps (**d**–**f**).

For noisy interferograms, especially for speckle fringes, we suggest using Windowed Fourier Filtering proposed by Qian et.al.^32,33^ to refine the wrapped phase before phase unwrapping. Figure 12 shows the filtered wrapped phases of the three methods and the corresponding phase extraction error map.

In the above specified situations, proposed APLEF method exhibits higher phase extraction accuracy. Next, we’d like to discuss the performance of APLEF under different variables.

**Figure 13:**
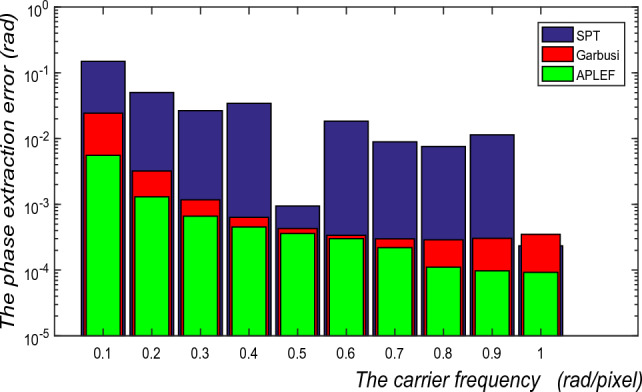
The phase extraction error of SPT, Garbusi’s method and APLEF versus the carrier frequency.

Firstly, the carrier frequency is of interested, for PLEF proposed in^24^ we know the carrier should be higher than 0.4 *rad/pixel* to ensure the Lissajous figure is higher than a quarter of an ellipse. Here, we assume the encoded phase is a defocus with PV of 2rad and the carrier frequency varies from 0.1*rad/pixel* to 1*rad/pixel*. Figure 13 shows the corresponding phase extraction error (RMS values) of the three methods, which clearly shows the better performance of APLEF.

**Figure 14:**
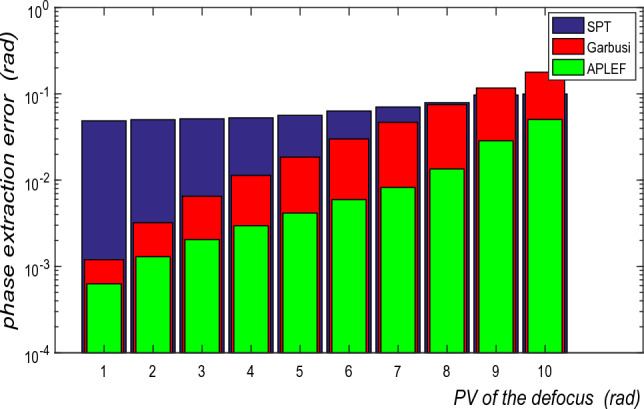
The phase extraction error of SPT, Garbusi’s method and APLEF versus PV of the defocus ( local phase shift error).

Secondly, we set the carrier frequency to 0.2rad/pixel and let PV of the encoded defocus various from 1rad to 10 rad. As we see from Fig. 14 with the increasing of defocus, the spatial domain methods will increasingly loss accuracy because the gradient of local phase is increased so the local phase shift error is increased. APLEF method however is more tolerant to local phase shift error than Garbusi’s method, which has also been demonstrated in Fig. 4.

**Figure 15:**
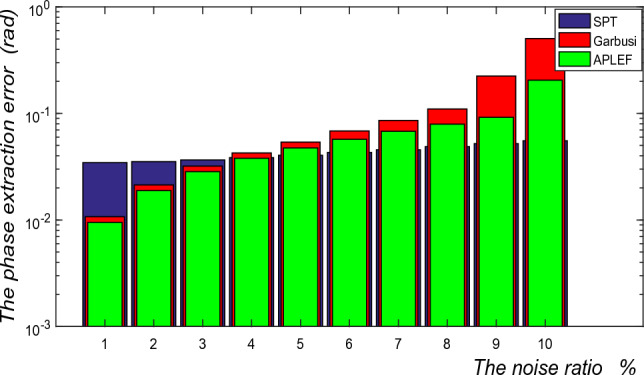
The phase extraction error of SPT, Garbusi’s method and APLEF versus different noise ratio.

Finally, the influence exerted by additive noise is estimated in Fig. 15. Although APLEF is more tolerant to additive noise than PLEF method, as a point-wise spatial method it is still very sensitive to additive noise comparing with the frequency domain method SPT. Therefore, it is crucial to perform the fringe pre-filtering before using APLEF for phase demodulation.

For fair comparation, we’d like to point out that as a spatial domain method both Garbusi’s method and proposed APLEF cost more time than SPT when the fringe direction map is out of consideration, especially for APLEF, which needs ellipse fitting process at every pixel of the interferogram. However, it is possible for parallel computing in dynamic circumstance as we already explained in the former PLEF method^24^.

## Experiments

**Figure 16:**
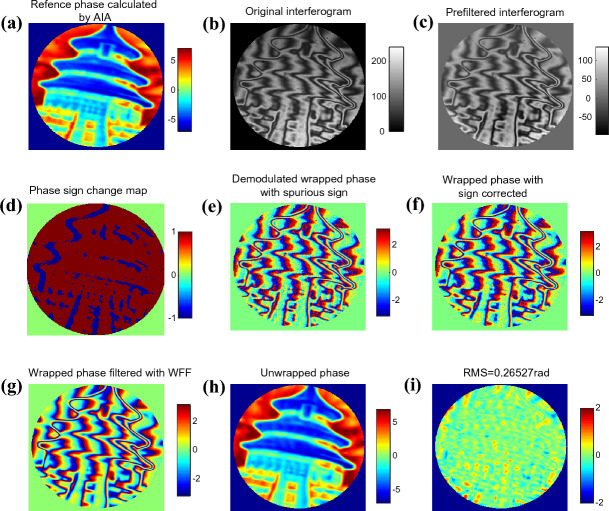
The reference phase calculated by AIA (**a**), one of the initial interferogram (**b**), pre-filtered interferogram (**c**), the phase sign change map estimated using the rference phase (**d**), the demodulated wrapped phases with sign ambiguous of APLEF (**e**), wrapped phase with sign corrected of APLEF,(**f**), the refined wrapped phase using WFF (**g**), the unwrapped phase with tilt removal of APLEF (**h**), the phase extraction error map of APLEF (**i**).

**Figure 17:**
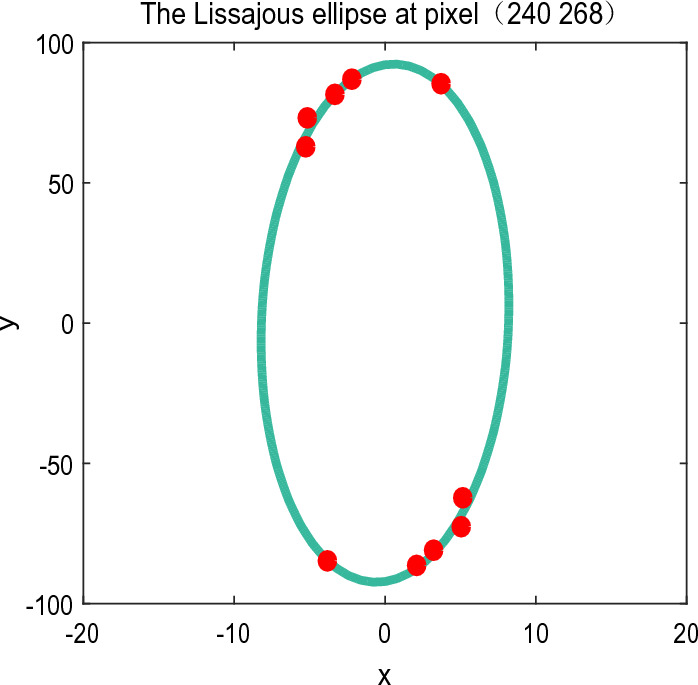
The created Lissajous ellipse at pixel (240,268).

The specimen is a flat mirror with the 3-D information of Temple of Heaven on its’ surface. We captured 13 phase shifted interferograms of the mirror using a Fizeua interferometer. The phase calculated by advanced iterative algorithm (AIA)^34^ is shown in Fig. 16a. The PV is 16.51 rad and it serves as the reference hereafter. Figure 16b is one of the captured inteterferograms, from which we can see the fringe pattern modulated by the 3-D information of Temple of Heaven is very complicated, high frequency fringes and low frequency fringes and even closed fringes are mixed together. The background is also highly non-uniform in spatial. According to proposed method, we used a Gaussian high pass filter with sigma=2 to filter the background term, then a *medfilter2* function with the window size of 3*3 is used to filter the high frequency noise. Fig. 16c shows the pre-filtered interferogram. Since closed fringes exist, we should estimate the phase sign change map first, the proposed swin-transformer based semantic segmentation net is used for estimation. The phase sign change map is shown in Fig. 16d. Figure 16e displays the demodulated wrapped phase of APLEF; we can notice the spurious phase sign around the closed fringes. With the help of phase sign change map, the ambiguous phase sign problem can be easily corrected, which is shown in Fig. 16f. WFF is used to refine the wrapped phase before the phase unwrapping process. Figure 16g is the refined wrapped phase. The final unwrapped phase with tilt removal is shown in Fig. 16h and the phase residual error map is exhibited in Fig. 16i. To show how the Lissajous ellipse is created during the phase demodulation process, we plot the Lissajous figure at pixel (240,268) for exhibition, which is shown in Fig. 17. From the above figures we see that, with the help of pre-filtering and post-filtering process, APLEF is able to extract the ground true phase from only one single interfrogram accurately; the RMS value of the phase extraction error is 0.265 rad which is only 6.9% comparing with the multi-frame algorithm AIA.

## Conclusions

We have proposed advanced pixel-level Lissajous ellipse fitting method for demodulation of single interferogram without carrier in this paper. It is free of fringe normalization and no need for careful scanning map. The demodulation process of proposed method and influence factors are discussed thoroughly through simulations. For a straightforward comparison, two respective methods, i.e., frequency domain method SPT and Garbusi’s spatial domain method are also employed simultaneously. The comparison indicates that proposed APLEF method exhibits better perfomance. The experimental application finally corroborated the effectiveness of proposed method.

## Data Availability

The datasets used and analyzed during the current study are available from the corresponding author on reasonable request.
